# Fostering Students’ Well-Being: The Mediating Role of Teacher Interpersonal Behavior and Student-Teacher Relationships

**DOI:** 10.3389/fpsyg.2021.796728

**Published:** 2022-01-04

**Authors:** Fang Zheng

**Affiliations:** ^1^School of Humanities, Tongji University, Shanghai, China; ^2^College of Information, Shanghai Ocean University, Shanghai, China

**Keywords:** student-teacher relationships, student’ well-being, teacher interpersonal behavior, learner-teacher relations, learners’ education

## Abstract

Well-being has become extensively viewed as apprehension for administrations in the last decades and schools have been progressively realized as locations for encouraging well-being which is a considerable development in inquiries on mediations connected to learner well-being. In this way, the function of teachers has got specific consideration regarding students’ well-being, given the merits of teacher-student interactions. High-quality educator-learner relationships offer a support base for long-term learners’ education. Educator interpersonal behavior that makes learners feel supported and cared for is known as emotional support. These behaviors can help learners’ emotional and social needs; meet learners’ families, and being available when learners need additional help. This review attempts to consider the eminence of teacher interpersonal behavior and learner-teacher relations in the classroom and indeed illustrate their relationship and influence on students’ well-being. As a final point, this review can provide suggestions and recommendations for teaching participants in the scholastic context.

## Introduction

With the arrival of positive psychology (PP), a relatively new subdivision of general psychology, studies have greatly emphasized the strengths, assets, and capabilities of individuals that lead to ultimate functioning and with this move, the focus has shifted from illness to wellness (Seligman, 2011). Positive psychology accentuated optimal functioning concerning physical, social, mental, and emotional well-being (Wang et al., 2021) and to the positive traits of people, constructive sensations and feelings, and the positive function of the environment and institutions in the growth of an individual’s well-being (Seligman and Csikszentmihalyi, 2014). In this way, PP seeks not only to improve participants’ well-being, but also to enhance their success, meaning, fulfillment, and resilience in all areas of life, particularly academics (Prior, 2019). The purpose of PP is to develop positive feelings, nurturing compassionate and helpful relationships, and establish a sense of perseverance in life, instead of coping with challenges (Seligman, 2011). Seligman and Csikszentmihalyi (2014) support this claim, proposing that psychology should turn its attention from the analysis and solution of difficulties to the expression of emotions, like love, optimism, and fulfillment, which are human values. The term learner well-being has been characterized in numerous ways, but in psychology, it is most often operated through measures of subjective well-being, positive attributes/character strong points, and the absence of psychological stress, like sorrow, worry, and anxiety (Kern et al., 2015). Well-being, in recent years, has become an obvious instructive goal in numerous educational organizations and it is understood that learners’ well-being should be a major result of public education (Opre et al., 2018). According to Mercer et al. (2018), positive learning places well-being at the center of educational practices along with educational content, which does not conflict with each other. Learners’ well-being is a multifaceted construct that includes the quality of school conditions in addition to a constructive emotive, subjective, and cognitive assessment of the reality of the school (Scrimin et al., 2016). Learners’ well-being is regarded as an emotional state (Holfve-Sabel, 2014) that is affected by various factors not only inside but also outside the classroom and the well-being of learners has a positive effect on their learning cycle and results. The well-being of learners enables them to cope with negative environmental impacts and those learners satisfied with their school develop positive attitudes towards the learning-teaching cycle and enhance their performance (Jarvela, 2011). Learners’ well-being offers a basis for succeeding socialization of adolescents into adulthood and is connected to superior socio-emotional operative and more positive self-assessment (Tuominen-Soini et al., 2012). The quality of education is measured by the well-being of learners and as a tool of measurement, educational success is the most important factor in conventional educational performance evaluation (Van Petegem et al., 2008). Students characterize their well-being as being safe, happy, respected, loved, and healthy (Anderson and Graham, 2016). Therefore, educational institutions must nurture and cultivate more emotional well-being to guarantee that they empower their students to become future productive citizens (Schonert-Reichl, 2017). The well-being of learners must be recognized in educational institutions that include their emotive, societal, physical, and intellectual state (Anderson and Graham, 2016). The classroom situation is a crucial situation for encouraging learners’ well-being so it should be a safe, comprehensive, humble, and caring learning milieu (Schonert-Reichl, 2017). The origin of societal provision and support can play a vital role in the well-being of learners, as an abundant number of studies have discovered fluctuating degrees of association between maintenance and well-being, reliant on the origin. When all guardians, peers, and educators were considered, support from educators demonstrated the strongest association with learners’ well-being (Danielsen et al., 2009).

The predictors of learner well-being are learners’ traits and motivations for going to school, their perceptions of educators’ interpersonal behavior, and school performance (Van Petegem et al., 2008). Teacher relational behavior has a significant role in reducing and diminishing the upshot of an education setting, other proficiency-based or less competence founded on learners’ inherent enthusiasm (Misbah et al., 2015), and in addition, it had an impression on learners. It is the fundamental issue for the learning achievement both experience and consequences as illuminated by Aldhafiri (2015) in his investigation that educators who could handle and regulate a good connection with their learners had the unmediated impression and could expand learners’ learning accomplishment. Teachers-students’ interpersonal behaviors could be formed from the determined contract. Teachers’ positive relational and interactional behavior can be verbal or non-verbal. Affection, proximity, stroke, credibility, affirmation, clearness, interpersonal friendship to learners, hilarity, and compliment are examples of positive communication behavior on the part of the teacher (Frisby, 2019). All of these behaviors encourage and stimulate successful interactions between educators and learners, resulting in a lively classroom, and satisfy students’ need for emotional and interpersonal support (Pishghadam et al., 2021). These kinds of behaviors satisfy learners’ rhetorical, relational, and emotional needs and want (Goldman et al., 2016).

Numerous researchers have mentioned that the educator’s personality, motivation, awareness, and passion are vitally important in the teaching process and in creating a positive teaching climate. The role of teachers is to comfort, guide, and support adolescents (Wentzel, 2016). Educators may be able to get along better with students since they devote so much time in class. Because of this, educators play a more integral role in the lives of students (Pianta et al., 2003). Educators who have the knowledge and abilities to build positive rapport with learners can be considered one of the most significant sources for structuring learners’ development (Ożańska-Ponikwia, 2017). It has been established that learning is essentially a social activity (Xie and Derakhshan, 2021) and since the time of Plato and Socrates, the relationship between educator and learner has been an area of concentrated study (Violanti et al., 2018). Learner-educator connections can be well-defined as essential and significant expressive and interpersonal relations that can be developed between learners and their educators as a magnitude of persistent and long-lasting communications (Longobardi et al., 2016; Wang and Guan, 2020).

The educator-learner relationship mirrors the closeness that results from an interaction between the two that is generally defined by mutual respect, trust, warmth, and little conflict (Aldrup et al., 2018). This relationship is critical to learner development as it makes learners feel safe and connected to their educators, makes them feel valued and supported by their educators, and ultimately motivates them to achieve positive results from learning in the cognitive, affective, and behavioral fields (Kunter et al., 2013). However, the connection between educator-learner relationships and the psychological well-being of the educator is often ignored. This is based on the fact that this factor is rarely studied, which is surprising since building relationships with learners is one of the primary responsibilities of the educator (Klassen et al., 2012). This is undoubtedly amazing since constructing associations with students is a portion of vital task in teaching (Van der Want et al., 2015). As part of the educational process, learners and educators have an equal role when it emanates to defining the success of the process (Delos Reyes and Torio, 2020). Positive educator-learner associations were theorized in various theoretical approaches such as attachment theory, self-determination theory (SDT), and sociocultural viewpoints (Davis, 2003). However, based on the literature review, most educator-learner association qualities have their origins in attachment theory in which the positive educator-learner connection may provide students with the safe, secure strengthening required to be involved in public events (Liu et al., 2015). Indeed, a strong rapport between the educator and the learner enhances learners’ feelings of security, which helps accelerate their ability to cope with adverse situations (Hughes, 2011).

In the same vein, Zimmer-Gembeck et al. (2006) found that strong social interactions can help learners to feel autonomous, engaged, and confident, motivating them to be interested in their educational objectives and to be enthusiastic about them. The SDT agenda proposes that the eminence of one’s well-being is affected by fulfilling three rudimentary core needs that are being encountered: the necessity for relatedness, competence, and autonomy (Patrick et al., 2007). SDT identifies the prominence of emotion associated with others as a rudimentary psychosomatic need and is reflected as a major component for working at ideal points (Ryan and Deci, 2017).

In addition, educator interpersonal behavior has been shown to influence learners’ success in addition to learner motivation, educational achievement, and happiness (Hill and Epps, 2010). In terms of learners’ well-being and academic outcomes, the interpersonal behavior of an educator has the potential to affect the educational process (Ryan and Deci, 2001). Learners can create a secure foundation in the educational setting through positive, high-quality relationships between them and their educators, which often enables them to explore, participate, and learn academic material more effectively (McCormick et al., 2013). The constructive connection between teacher-student relationships and learners’ social-emotive well-being has also been frequently deep-rooted because how teachers treat students affects how students treat each other (Waldinger et al., 2015; Chory and Offstein, 2018; Dietrich and Cohen, 2019). Although the above-mentioned issue has been inspected in countless situations and regions, based on the researcher’s information, on the one hand, in comparison with educators’ well-being, learners’ well-being is a less explored topic and on the other hand, teacher behavior and student-teacher relation have undergone an unexplored part expecting additional investigation.

## Reviewing the Related Literature

### Concept of Well-Being

The goal of positive psychology is to promote subjective well-being and happiness. By gathering positive information about well-being, positive psychologists aim to enhance psychological functioning and individual success (e.g., enhancing subjective well-being) (Seligman, 2011). As a part of PP, well-being is defined as constructive and enduring attributes that allow people and communities to prosper and develop. Indeed, well-being is well-defined from two viewpoints such as hedonic or eudemonic (Ryan and Deci, 2001). From the former point of view (hedonic), the term subjective well-being (SWB) is employed in numerous inquiries, dealing mainly with the virtual constructive and destructive emotional assessment of the individual in their living environment (Kim-Prieto et al., 2005). SWB is a key phrase that is used to explain the well-being that individuals experience along with the subjective assessment of their life (Diener and Ryan, 2009). In comparison, eudaemonic explanations of well-being naturally encompass meaning and self-actualization, explaining well-being as the extent to which an individual is completely functional (Ryan and Deci, 2001). Both concepts emphasize a nous of positivity; nevertheless, the hedonic viewpoint focuses on the feelings, satisfaction, and pleasure of the individual at a given moment, while the eudemonic viewpoint is more shaped by ideas of a life well lived that is incorporated in societal settings and societies (Disabato et al., 2016). Student well-being must be accepted in learning situations as containing learners’ emotional, social, physical, and mental routes of being that it fluctuates everyday as it is influenced by on whether their emotional, social, physical, and mental desires have been gratified (van der Kaap-Deeder et al., 2017). The concept of well-being offered by Seligman (2011) consists of five components, with the PERMA model being the newest. In this new theory, Seligman proposed that the key to well-being is nurturing a variety of elements, including good feelings, meaningful relationships, accomplishments, bonds, and commitment. To achieve well-being, we need to consider the following.

#### Positive Emotions

Good feelings are one of the most powerful motivators in human behavior. Reading, traveling, and engaging in activities that make individuals feel happy and motivate them to do things in the best way. Aspirations and dreams for the future are nurtured by good feelings (Oxford, 2016). Emotional well-being can also improve job performance, promote physical wellness, and enhance interpersonal quality (Wang and Guan, 2020; Wang et al., 2021).

#### Engagement

In this context, engagement is the degree of commitment, participation, and focus. It also refers to the level of enthusiasm shown for pursuits such as recreation or interests (Derakhshan, 2021). Flow is a key concept, which describes losing the self-concept during a moment of stillness and concentrating solely on the present. The science of positive psychological theory defines the conception of ‘flow’ as a state of full commitment in the present moment. Flow occurs when we are fully present, engaged in what we enjoy, and fully engaged in the present moment (Seligman, 2011).

#### Relationships

The need to feel close to others, to love them, to experience physical and psychological connection with them is a strong inner need. Having meaningful relationships with our peers, relatives, and coworkers ensures our well-being. A sense of belonging can be created by relationships that are supportive or negative, such as close relationships with friends and relatives or poor relationships with (Sandstrom and Dunn, 2014).

#### Meaning and Purpose

Wisdom of meaning and purpose refers to using resources to complete important goals, instead of for the self. A time commitment to something more important than ourselves can boost our performance. You can do this through volunteering, becoming a member of a community club or social or spiritual organization, or by learning for a particular purpose. The activities listed above have a well-defined purpose and giving people a strong reason for participating.

#### Accomplishment

It refers to living a successful, purposeful life. The goal of this approach is to value it for its reasons, regardless of whether it brings pleasant feelings, significance, or any supportive connections (Seligman, 2011).

### The Teacher-Student Relationship and Interpersonal Behavior

Hypothesized as the extent of friendship and dispute inside this association, the educator-learner association, and rapport play a vital role in the learners’ adjustment to the school (Zee et al., 2013). Building resilient and caring interactions with educators enable learners to be more self-confident, protected, and knowledgeable in the educational environment. Moreover, it enables and accelerates positive associations with peers and is associated with higher educational performance (Olivier and Archambault, 2017). Pianta et al. (2003) proposed the interpersonal relationship between educators and learners as a notion of an educator-learner affiliation. This can be regarded as the educators’ awareness of their connection with learners, which encompasses three dimensions, that is, friendship, struggle, and dependence. The closeness (friendship) dimension can mirror warmth, directness, and safety in the educator-learner connection (O’Connor, 2008). The conflict dimension mirrors the degree to which educators perceive the connection as bad, unpredictable, contradictory, and uncomfortable. Ultimately, the development of learners in their dependence on others refers to the dependency dimension (Pianta et al., 2003). Indeed, The educator-learner conflict has been regarded as one of the most significant characteristics of the educator-student rapport, influencing the adaptation of learners in the classroom (Han and Wang, 2021). Educators, in their relationship with learners, are inclined to work as an authority reinforcing suitable behavior and modifying inappropriate engagements. They also tend to function as originators of societal communications and constructive associations with students (Quaglia et al., 2013).

Of these three dimensions, the closeness in the relationship between educators and learners is the most desirable. Furthermore, it is also mentioned that closeness (friendship) is the sole positive dimension of the connection between educators and learners since instructing is an action that is imbued with positive feelings (Milatz et al., 2015). Studies indicate that a favorable educator-learner rapport, marked by good communication, help, and personal connections, minimizes learners’ tension and reduces the signs of intrinsic and extrinsic behaviors (Longobardi et al., 2016), and let down their stages of affecting and uttering indicators. Psychological symptoms can be perceived as indicators of emotional well-being in learners, and they impact a learner’s adaptation to the educational environment. A depressed-nervous state accompanied by emotional withdrawal is a sign of intrinsic behaviors, whereas violent and hyperactive behavior indicates extrinsic behaviors. Teachers have considerable duty in guaranteeing that these relationships are formed and then remain to be a cause of support for their scholars. Schonert-Reichl (2017) acknowledged that a teacher’s own capability forms the nature of the association they have with their students so teacher-student relationships influence student well-being since the daily intimacy the two groups share impacts whether their needs have been known and somewhat fulfilled.

Playing a significant function in certifying well-being and bonding in the teaching space is the interaction between educators and learners. Educators have the crucial accountability for engaging learners in-class actions and setting the situations and sense of the classroom ambiance. As opposed to a dominant or disconnected educator, a receptive and approachable one can make students feel like they are in class with positive affection. Conflictive attitudes of educators have been demonstrated to have destructive emotional and instructive impacts on learners (Sava, 2002), while compassionate relations with educators are related to higher learner gratification with the school.

### Theoretical Backgrounds Around Teacher-Student Relationship

Attachment theory and self-determination theory are two prevalent theories in the social-psychological field that emphasize the significance of high-quality educator-learner relationships (Verschueren, 2015).

#### Attachment Theory

The attachment theory highlights the significance of affective dimensions in defining high-quality educator-learner relationships (Verschueren, 2015). The attachment theory has been specifically utilized as a system to suggest three significant aspects to examine the emotional excellence of educator-learner associations, namely, closeness, conflict, and dependency (Pianta et al., 2003). Closeness alludes to the security and warmth that is felt in a connection and characterizes interactions through open communication, in which learners feel easy and calm enough to seek support from their educators. The resistance and negativity in relationships with educators are known as conflict. When there are high levels of conflict in relationships, inharmonious interactions and the absence of rapport lead to high levels of friction. Possessiveness and overdependence on an educator are known as dependency and are believed to decrease learners’ consideration of the school setting and other societal connections (García-Moya, 2020). The presentation of this notion to the school environment explicates the influence of high-quality associations on learners’ learning enthusiasm and success in terms of the point that constructive and expressively compulsory educator-learner relationships offer learners the security they require to have a well educational curiosity and commitment to scholarship assignments (Carmona-Halty et al., 2019). Because of the quality of their relationship, educators offer their learners vital psychological assets such as hope, competence, resilience, and optimism (Carmona-Halty et al., 2019).

#### Self-Determination Theory

Self-determination theory is an instruction hypothesis of students’ social growth and motivation widely used by educational psychologists that have an interest in well-being and motivation. The extent of SDT is large and includes sub-theories. The overview presented in this review takes the theory of basic needs into consideration (Reeve, 2013). The straightforward requirements theory highlights the significance of three rudimentary and global emotional needs, that is, relatedness, autonomy, and competence (Ryan and Deci, 2017). The need to develop close social relationships with others is known as relatedness and was originally characterized as the need to feel belonging and being connected to others (Klassen et al., 2012). Furthermore, it has been utilized interchangeably with numerous similar constructs, like connectedness. Autonomy alludes to a feeling of independence of special and governor over one’s conduct. Regarded as significant characteristics of autonomy were an inner locus of connectedness, a feeling of psychological freedom, and perceived freedom of action (Reeve, 2013). The requirement for autonomy must not be misinterpreted as a need for total freedom or objectivity; rather, relatedness and autonomy are corresponding needs (Ryan and Deci, 2001). Ultimately, competence alludes to the requirement to be effective in the performance of individuals’ obligations and to be competent, and to exhibit knowledge of the actions that a person performs. Self-determination theory holds that people will be enthusiastic and emotionally well-adjusted if they have numerous chances to meet these three basic needs (Ryan and Deci, 2001). To boost learners’ well-being, educational settings that satisfy their needs for relationship, skill, and independence are essential.

## Implications and Future Directions

The present review is noteworthy for teachers as their consciousness of the relations of the eminence of educator–learner interactions with learners’ passions may increase the quality of these connections. An encouraging connection with learners has the prospective to notify educators about learners’ requirements, wants, and desires that indeed enable learners to deal positively with tasks, consequently enhancing their well-being. Educator-learner relations are characterized by the closeness that arises from their interaction. It is generally recognized that the rapport between educator and learner is marked by a sense of trust, reciprocity, friendship, and a lack of disagreement (Aldrup et al., 2018). Such a rapport is important for learners’ success because it establishes a sense of safety and attachment to educators, as well as feelings of respect and encouragement from educators, and thus, learners are eager to accomplish intellectual, behavioral, and emotional educational objectives (Rafsanjani et al., 2019). As learners’ well-being develops, likely, the learning environment, educators’ attitudes and methods, and the communication between learners and educators should be taken into account. The well-being of learners is enhanced when the educator-learner rapport is positive. The well-being of learners suffers when in a conflictual interaction. It underscores the importance of building a positive rapport between educators and learners. Various authors have emphasized the importance of social relationships for academic achievement in educational settings based on the fact that the teacher’s behavior directly influences learners. Relations between educators and learners are a continuous process, and this process determines their manner of interaction. In the educational setting, learners’ feelings of relationship and community can be enhanced if their educator has a close rapport with them, which may support positive attitudes and inhibit negative ones. It is plausible that learners are satisfied with their primary emotional needs for belonging in a positive educator-learner rapport, while educators are more responsive to learners’ specific learning requirements. Positive engagement in schoolwork may help teenagers develop interest and enthusiasm, which is likely to enhance their educational presentation. Alternatively, the need for a close relationship is seldom satisfied in the context of a conflict-ridden educator-learner rapport. Accordingly, learners feel less engaged in education and feel more stressed and depressed. Interactions and their levels are essential for not only teachers but also learners so they should be taken into granted. For the students to accomplish high degrees of well-being, there must be a supportive bond between them.

The role of educators in affecting the well-being of learners is indisputable and they can take on different roles, like watching their conversations, being careful with the feedback to learners, paying attention to them, asking questions to involve them, and reconsidering the classroom supervision to control the connections (Mercer and Dörnyei, 2020). Educators must create a safe, compassionate, and humble class where learners are truly heard. They must understand that their well-being has a flow effect on their learners. Therefore, educators must also cultivate their wellness ability to do their occupation successfully. The best way for educators to engage learners is to establish a dominate-cooperative learning model that allows autonomy while ensuring discipline when necessary. To manage and develop learners’ well-being effectively in the education setting, educators need to participate in training and professional advancement consistently. In addition, by controlling anxiety, dealing with conflict, managing social interactions, developing strong relationships with their children, and being engaged in all of the decisions they make, educators can empower learners to develop and enhance their well-being. Despite the support of friends and guardians, this research proposes that educators might play the most effective role in the well-being of learners in early and middle adolescence at school. As declared by Huebner et al. (2009), learner well-being has been demonstrated to be connected with a variety of significant school results, like grades, classroom behavior, and early school leaving. Therefore, educators must concentrate on stimulating the well-being and educational achievement of learners to prepare them for achievements. In school life, educators’ social support may be indirectly connected with school-related subjective well-being of learners through the psychosocial process of meeting three fundamental psychosomatic requirements in school. Educators should be encouraged not only to concentrate on the extent that societal provision provided by peers and educators satisfies the basic psychological needs of adolescents at school, but also to construct societal maintenance consistent with these needs, and consequently, contribute to the enhancement of learners’ subjective well-being related to the school and their progressive change.

A good connection with the educator can let learners express their emotions and worries openly, evoke suitable guidance from educators, and promote successful interactions in the classroom. Close relationships with educators can provide learners emotive provision and security, which sequentially can reinforce constructive manners and rule out more destructive behaviors in the class and with their friends outside the class. Learners experience emotive and attitudinal firmness and receive satisfying passionate support from their educators by associating with them, leading to effective learning.

Taking into account the interaction between learner-educator relations and learners’ and educators’ traits have obvious consequences for planning programs and policies. Professional development programs are essential to ensuring that educators are conscious of the inherent bias in the learner-educator rapport, and they are taught to teachers to be aware of how to handle learner-educator rapport, and empower them to improve interactions between learners and educators that lead to students’ well-being, as well. School professionals should therefore consider making systematic and ongoing endeavors to promote and oversee a progressive school environment and school-connected subjective well-being of learners. Curriculum designers are expected to reflect successful educator-learner relationships as the foundation for learning, designing assignments and activities in such a way as to maintain negotiations and associations between educators and learners to take on assignments and engage them. Nonetheless, there is a dearth of investigations on how positive educator behavior can be improved in training courses to efficiently engage learners. Forthcoming studies must examine causal relationships by introducing classroom mediations and treatments aimed at enhancing educator-learner relationships. Employing interventions or teachings is suggested in enlightening teachers’ relationships with students as it fosters students’ well-being.

## Author Contributions

The author confirms being the sole contributor of this work and has approved it for publication.

## Conflict of Interest

The author declares that the research was conducted in the absence of any commercial or financial relationships that could be construed as a potential conflict of interest.

## Publisher’s Note

All claims expressed in this article are solely those of the authors and do not necessarily represent those of their affiliated organizations, or those of the publisher, the editors and the reviewers. Any product that may be evaluated in this article, or claim that may be made by its manufacturer, is not guaranteed or endorsed by the publisher.
